# Comparative analysis of dosimetry and predictive somatotype parameters of prone and supine whole-breast irradiation among Chinese women after breast-conserving surgery

**DOI:** 10.3389/fonc.2022.1011805

**Published:** 2022-11-17

**Authors:** Yi Gao, Li Wang, Han Bai, Xiang Pan, Lan Li, Li Chang, Yaoxiong Xia, Wenhui Li, Yu Hou

**Affiliations:** Department of Radiation Oncology, The Third Affiliated Hospital of Kunming Medical University, Tumor Hospital of Yunnan Province, Kunming, Yunnan, China

**Keywords:** breast neoplasms, radiotherapy, prone position, somatotype, Chinese women

## Abstract

**Purpose:**

Finding a better treatment position (prone or supine) for whole-breast irradiation for Chinese female patients diagnosed with breast cancer by identify the associations between predictive somatotype parameters and dosimetric gains.

**Materials and methods:**

Two volumetric-modulated arc therapy (VMAT) plans were deployed for whole-breast irradiation in supine and prone position with a total dose of 50 Gy in 25 fractions. Dose-volume parameters were compared and analysed both in the target volume and organs at risk, and equivalent uniform dose-based figure-of-merit (fEUD) models were further used to quantitatively evaluate the overall merits of the two plans. Body shape parameters, including body mass index (BMI), body surface area (BSA), breast shape, cup size, bust size and chest size, were collected. Anatomic features such as the central heart distance (CHD) were measured on supine CT. Spearman’s correlation analysis, receiver operating characteristic (ROC) curve analysis, and the linear regression models were conducted.

**Results:**

Doses to the heart and left anterior descending coronary artery (LADCA) are greater in left-sided breast cancer (BC) patients in the prone position than in the supine position, and the opposite was true for right-sided BC patients (p<0.001). 19 of 63 patients (5 left-sided and 14 right-sided BC) achieved greater benefit from the prone position according to the fEUD score. Right-sided BC patients with a bust size ≥92.25 cm, drop-type breasts and cup size ≥B are very likely to benefit from prone-position radiotherapy. The CHD is significantly positively associated with △fEUD among right-sided BC patients (rho=0.506, p=0.004). Using a cut-off point of 2.215, the CHD had 71.4% sensitivity and 81.2% specificity in predicting a successful prone plan.

**Conclusions:**

Right-sided BC patients had better dosimetric gain in the prone position than left-sided BC patients. The CHD is an especially good and novel predictor that could help to select prone-benefitting right-sided BC patients.

## Introduction

Breast cancer (BC) is the most commonly diagnosed cancer both in China and the whole world (1, 2). Given the increased prevalence of cancer screening, the proportion of early BC diagnoses has significantly increased. Breast-conserving surgery (BCS) is the standard surgical treatment for operable, early-stage BC. The percentage of patients undergoing BCS increased from 10.83% to 30.83% between 2006 and 2015 in China (3). Adjuvant radiotherapy (RT) after BCS for early-stage BC can effectively improve the survival rate and reduce the risk of recurrence (4, 5) while providing satisfactory cosmetic results as well as psychological support. As such, postoperative RT is considered the standard treatment for early-stage BC.

Generally, the supine position has been widely used for clinical RT in BC, as it is more comfortable and reproducible for patients than the prone position. However, irradiation for BC patients in the prone position could achieve better dose distributions and spare more normal tissue than the supine position (6–8), especially those with large breasts. Two randomized trials focused on the 2-year and 5-year whole breast irradiation outcomes in the prone versus supine positions among large-breasted women (9, 10) demonstrated better cosmetic outcomes and lower rates of late toxicity in the prone position.

Consistent criteria have yet to be established for selecting patients who would benefit most from prone RT. Studies on prone positioning for BC treatment have mainly been conducted in American and European countries. One South Korean study (11) suggested that patients with small breast volumes (such as those with a clinical target volume (CTV) of approximately 100 cm^3^) could also benefit from the prone position.

Therefore, we conducted this study comparing the prone position with the supine position for delivering volumetric-modulated arc therapy (VMAT) to Chinese BC patients. The purpose was to assess the effects of the prone position on the dose distribution and determine differences in normal organ sparing between VMAT in the two positions. We further attempted to identify that body shape characteristics associated with prone position-benefitting breast RT among Chinese women to provide a reference basis for the rational, clinical use of the radiotherapy position.

## Materials and methods

### Patients and treatment simulations

The inclusion criteria were as follows: age between 18 and 70 years, pathologically confirmed stage 0-II BC (Tis-T2) after BCS, and Eastern Cooperative Oncology Group performance status 0 or 1. Patients were excluded if they needed irradiation of the locoregional lymph node area and had prosthetic implants, supraclavicular/internal mammary nodes, bilateral BC, previous irradiation or other malignancies. All patients were asked to provide their written informed consent before being registered in the study, and the present study was approved by the ethics committee of Tumor Hospital of Yunnan Province (approval number of Institutional Review Board: KYLX2022025).

**Figure 1:**
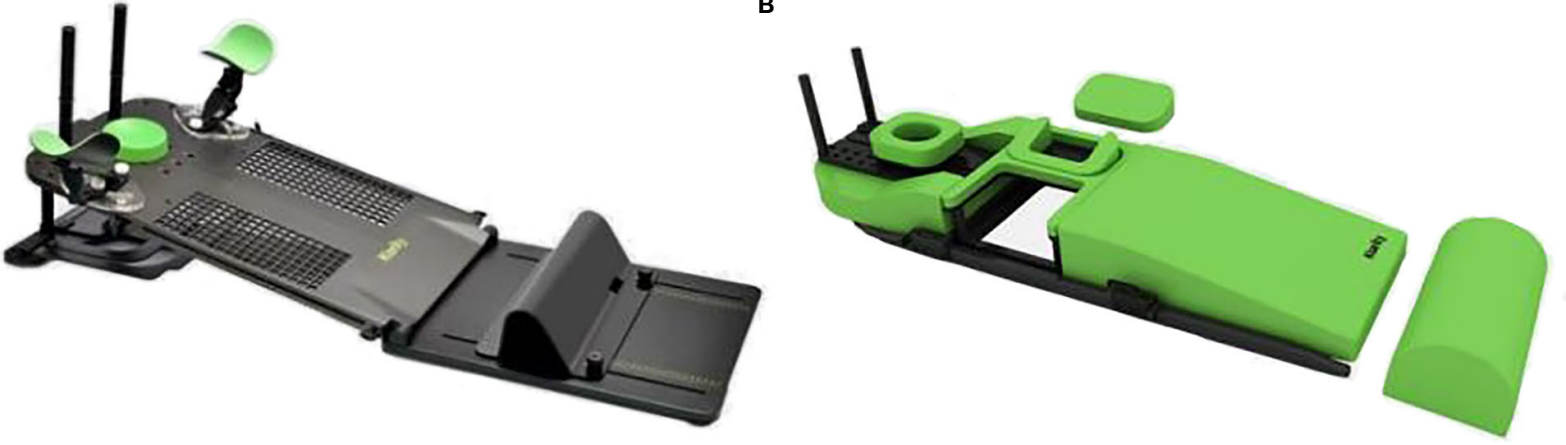
Supine/Prone breast board. **(A)** Supine breast board(Klarity, R610-DCF).**(B)** Prone breast board(Klarity, R62-BCF4).

Enrolled patients underwent two computed tomography (CT) simulations in the supine and prone positions. First, patients were imaged on a conventional supine breast board (R610-DCF, Klarity Medical & Equipment Co. Ltd. Guangzhou, China) with arms above the head to adequately expose the breast (**Figure 1A**). Then, they were repositioned on a prone board (R62-BCF4, Klarity Medical & Equipment Co. Ltd. Guangzhou, China) with a removable right and left aperture to allow the index breast tissue to hang away from the chest wall (**Figure 1B**). The borders of the breast tissue and midline of the chest were marked for each patient with radio-opaque wires before CT acquisition. For both setups, free-breathing CT scans were performed using a large-aperture CT system (SOMATOM Sensation Open 24, Siemens, Germany) without contrast, starting below the mandible and caudally ending below the lower edge of the liver with a slice thickness of 3.0 mm. The CT scan images were transferred to the Treatment Planning System (Monaco version 5.11, Elekta AB, Stockholm, Sweden) of the department.

### Radiotherapy planning and evaluation

**Figure 2:**
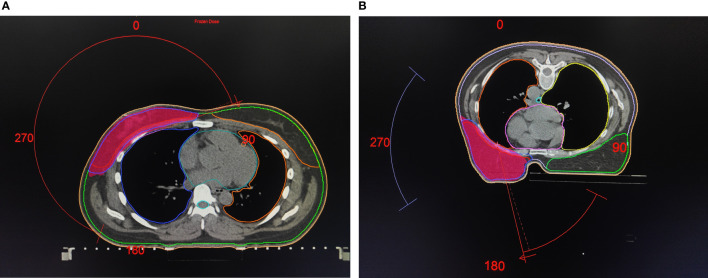
Supine/Prone treatment plans with target and organs at risk delineation. **(A)** Treatment plans of the supine position. **(B)** Treatment plans of the prone position.

CTVs and organs at risk (OARs) were contoured manually according to the Radiation Therapy Oncology Group (RTOG) breast cancer atlas (12) (**Figures 2A, B**). The breast CTV was contoured up to the inferior margin of the clavicular heads (cranially), to the farthest visible breast contour, at approximately the level of apex disappearance (caudally), to the perforating mammary vessels or to the edge of the sternum (medially), to the anterior edge of the latissimus dorsi (laterally), to the junction of the breast tissue and the pectoralis muscles (posteriorly), and up to 5 mm under the skin surface (anteriorly). The CTV was delineated based on the glandular breast tissue visible on the CT images. Planning target volumes (PTVs) were generated by the addition of three-dimensional, 5-mm margins to the CTV up to 5 mm from the skin. The whole heart was delineated in accordance with the guidelines proposed by Feng et al. (13). The left-anterior descending coronary artery (LADCA) does not include the left main trunk, which was delineated down to the apical level. Considering the planned volume of the heart while beating, the uniform diameter of the LADCA is 1 cm. OARs such as lungs, spinal cord, esophagus and liver were delineated according to the RTOG 1106 atlas (14). In detail, all inflated and collapsed, fibrotic, and emphysematic lungs were contoured with inclusion of small vessels extending beyond the hilar regions, excluding the proximal bronchial tree. The contralateral breast was delineated up to 5 mm under the skin surface. The spinal cord was delineated starting at the same cranial level as the esophagus to the bottom of L2 or at the level in which the cord ended. The oesophagus was delineated starting cranially from the inferior margin of the cricoid and ending inferiorly at the gastroesophageal junction. The whole liver was delineated along the outer edge of the liver, excluding the gallbladder. The CTV and OARs were delineated on CT slices by one radiation oncologist and verified by two other senior experienced radiation oncologists.

The RT plans were generated for a Versa HD linear accelerator (Elekta Medical Systems Co., Stockholm, Sweden) with 6 MV photon energy. Previous studies have showed that (15), VMAT could achieve better target conformability and uniformity compared to intensity-modulated radiation therapy (IMRT). Considering the further comparison of dosimetric differences between important normal organs, such as the heart and lung, on the basis of ensuring adequate target coverage, the VMAT irradiation technology being commonly used in our institutions and in this study. Referring to the correlational researches (15, 16), we used a continuous VMAT (cVMAT) treatment plan with one dual arc of (140.0 ± 10.0)∼(320.0 ± 10.0)° for the supine position (**Figure 2A**). The prone plans consisted of tangential VMAT (tVMAT) plans with two tangential dual arcs of (140.0 ± 10.0)∼(120.0 ± 10.0)° and (340.0 ± 10.0)∼(310.0 ± 10.0)° rotations, accounting for the limitations of the machine boom rotation (**Figure 2B**). A prescription dose of 50 Gy in 25 fractions was delivered to the whole breast according to the ICRU report 83 (17), with the prescribed dose covering ≥95% of the PTV and ≤7% receiving 105% of the prescribed dose. And according to the relevant research (11) and institutional experience, we constrainted OARs were as follow: V20 < 30% for contralateral lung; mean heart dose < 6 Gy (left and right), and maximum dose of spinal cord <40Gy in the supine position; V20 < 20% for contralateral lung; mean heart dose < 8 Gy (left) or 6Gy (right), and maximum dose of spinal cord < 40 Gy in the prone position. A radiotherapy planning consensus for both sets was achieved by the agreement of more than two physicists. Only the supine treatment plan was used for real-world clinical daily RT.

All plans were compared according to the planning target volume coverage, dose-volume histogram and other dosimetric parameters of normal tissues. For target coverage, we recorded the minimum, maximum and mean doses to the PTV (Dmin, Dmax, Dmean), V95%, V105%, V100%, homogeneity index (HI) (18), and conformity index (CI) (19). The CI and HI were calculated using the following equations: 1) CI=(TV95/TV) × (TV95/V95), where V95 is the total volume receiving 95% of the prescription dose, TV is the target volume, and TV95 is the target volume receiving 95% of the prescription dose, with values closer to 1 indicating optimal conformation; 2) HI= (D2% -D98%)/D50%, where D2%, D50% and D98% are the doses covering 2%, 50% and 98% of the volume of the PTV, with lower values indicating administration of a more homogeneous dose to the target volume. For normal organs, such as the heart and ipsilateral and contralateral lung, we compared Dmax, Dmean, and the percentage of the volume that received more than 5, 10, 20, 30, and 40 Gy (V5, V10, V20, 30, and V40).

### Anthropometric body shape parameters

**Figure 3:**
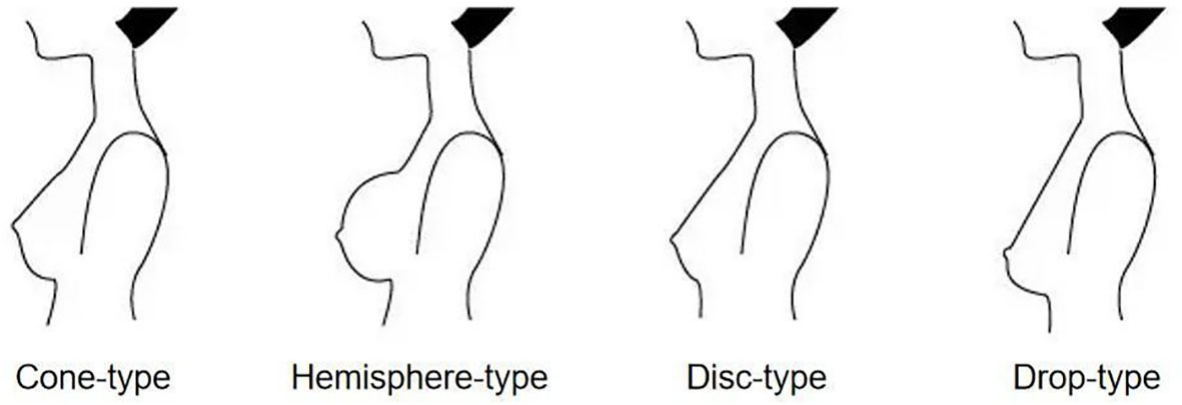
Type of breast shape.

Body shape parameters, including height, weight, body mass index (BMI), body surface area (BSA), bust size and chest size were collected. BMI=weight(kg)/height(m)^2^. BSA=0.0073×/height(m)+0.0127×weight(kg)-0.2106. Bust size was measured as the circumference around the chest at the plane of the nipple. Chest size was measured as the circumference around the chest under the fold of the breasts. We also collected general information, including the breast shape (**Figure 3**) and cup size of all patients.

### Supine anatomic feature measurements

Song et al. (20) reported that breast separation (BS) was positively correlated with the mean skin dose and was an important parameter for the selection of electronic tissue compensation radiotherapy. BS was defined as the distance between the entry points of two opposing beams on the central plane. In addition, the central lung distance (CLD) has been said to provide a close estimation of the volumetric lung dose; when the CLD is greater than 3.0 cm, the reduction in the dose delivered to the ipsilateral lung was found to be remarkable when using the medial breast technique (21). The CLD was defined as the perpendicular distance from the chest wall to the posterior border of the tangential fields.

**Figure 4:**
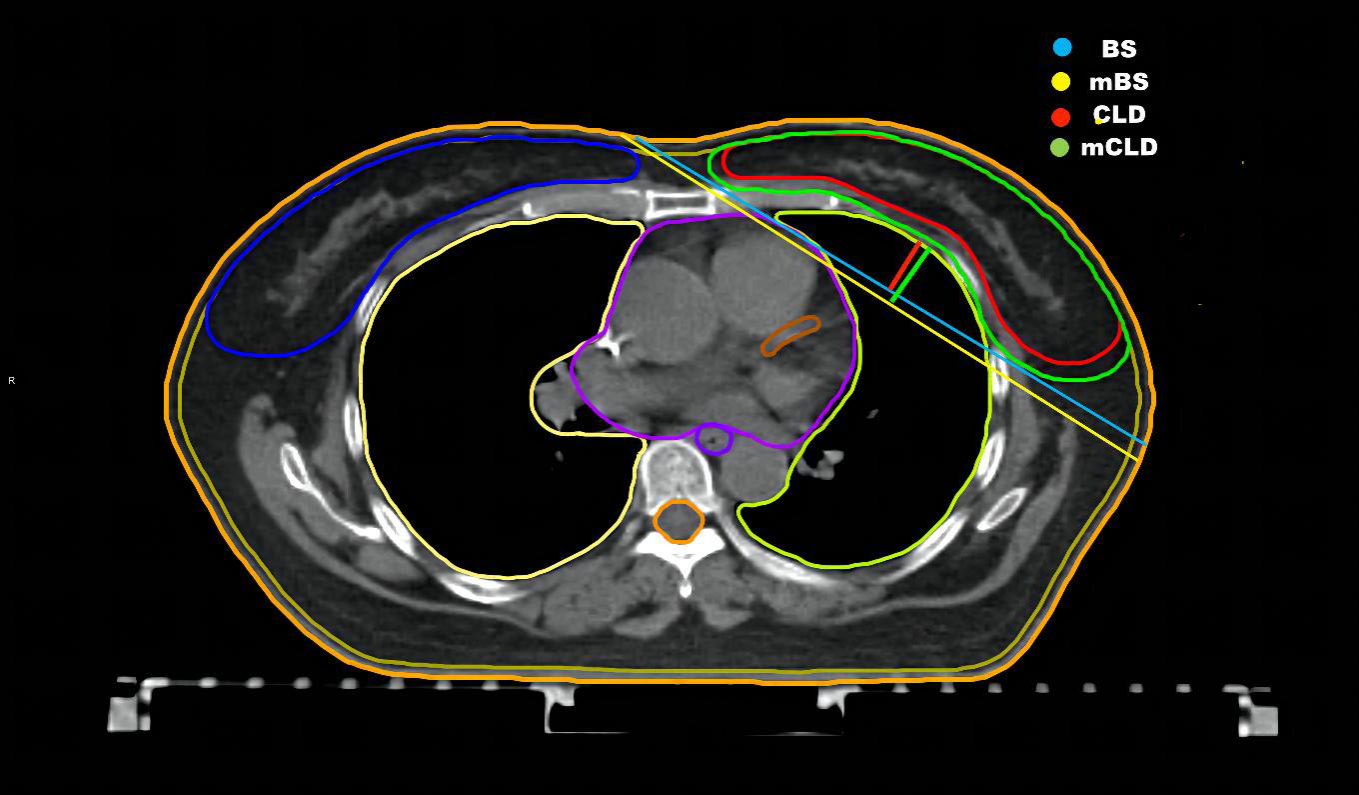
Anatomic parameters in the supine CT. The central plane from the low edge of clavicular head to the cardiac apex in the supine CT. The breast separation (BS) is the distance between entry points of two opposing beams on the central plane. The central lung distance (CLD) is the perpendicular distance from chest wall to the posterior boarder of the tangential fields.The modified breast separation (mBS) is the distance from the border of the sternum and the anterior border of latissimus dorsi then extending to skin.The modified central lung distance (mCLD) is the maximum perpendicular distance from BS to the posterior part of the anterior chest wall.

Since the BS and the CLD could only be recorded after RT planning, we choose the modified breast separation (mBS) and modified central lung distance (mCLD) as alternative indicators which could be measured on routine chest CT. The mBS was defined as the distance from the border of the sternum and the anterior border of the latissimus dorsi extending to the skin. The mCLD was defined as the maximum perpendicular distance from the mBS to the posterior part of the anterior chest wall. Both parameters were measured on the central plane (similar to the central PTV plane) from the lower edge of the clavicular head to the cardiac apex on supine CT (**Figure 4**).

**Figure 5:**
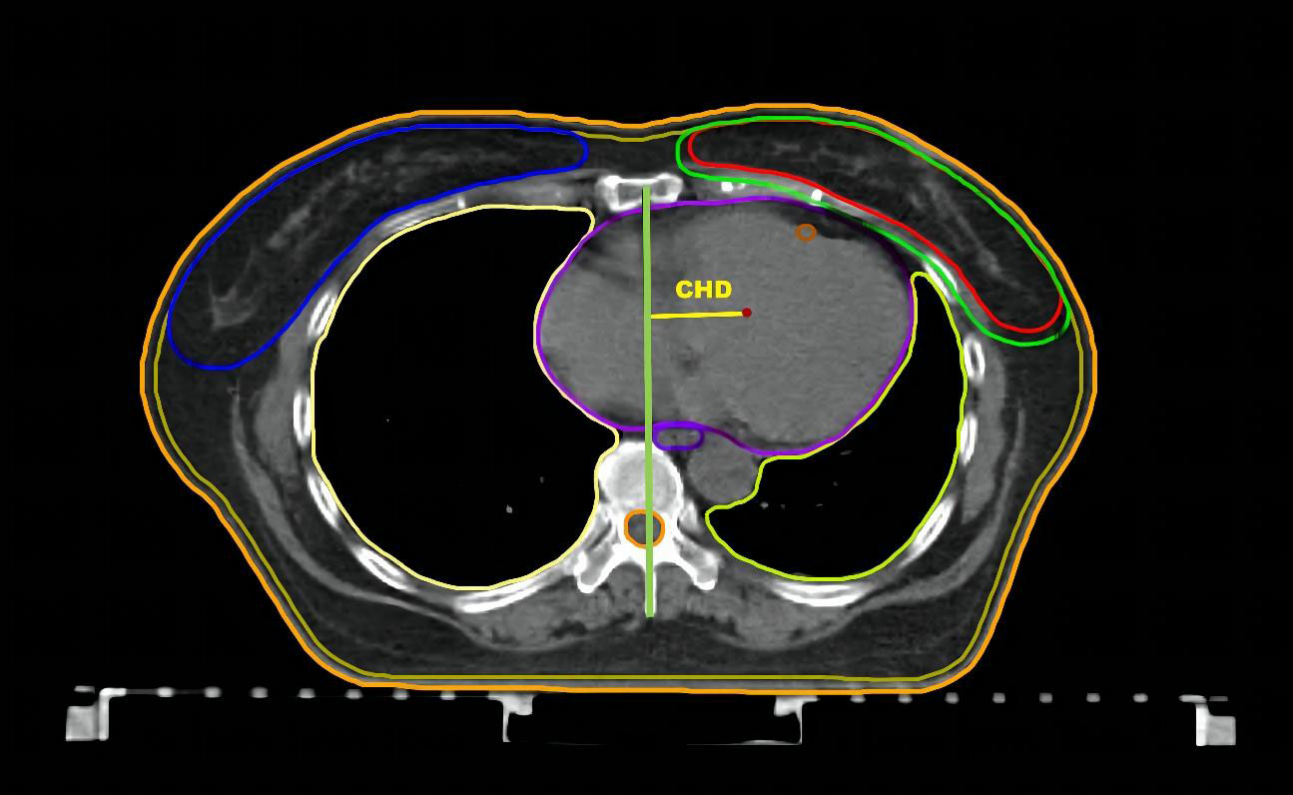
CHD in the supine CT. The central heart plane. The central heart distance (CHD) is the perpendicular distance from centre point of heart to the midline.

Additionally, we creatively assessed a new concept, the central heart distance (CHD), as a predictive parameter for the heart doses. The CHD is the perpendicular distance from the centre point of the heart to the midline on the central heart plane on supine CT (**Figure 5**). The central heart plane is the middle CT slice from the bifurcation of the pulmonary trunk (superior border) to the last slice containing cardiac tissue (inferior border). The midline was measured from the sternum centre to the posterior margin of the spinous process. The centre point of the heart was automatically computed as a three-dimensional point by Monaco^®^ TPS 5.11.

### EUD and fEUD models for plan comparison

The equivalent uniform dose (EUD), defined as the uniform dose giving the same biological effect as a given nonuniform dose distribution, was generalized to normal structures and tumours by Niemierko in 1999 (22). The generalized EUD (gEUD) was calculated based on the power-law dependence of the dose response for the tumour and the OARs with the following simplified formula: $\text{EUD}={\left(\sum_{i}\nu_{i}D_{i}^{a}\right)}^{1/a}$ , where vi is the fraction of the reference volume irradiated with dose Di, and a is a free structure-specific parameter that is usually positive for OARs and negative for tumours. Base on the article by a previously published article by Boughalia et al. (23), we set a(PTV)=-6, a(heart)=2, a(ipsilateral lung)=2, a(contralateral lung)=5, a(LADCA)=5, a(contralateral breast)=5, and a(liver)=5. The vi and di values in the prone and supine position plans of each patient were derived from the Monaco TPS and substituted into the EUD formula to calculate the EUD values of the target areas and OARs in the two plans.

Qi et al. (24) created an EUD-based figure-of-merit (fEUD) to quantify the overall plan quality when attempting to use the EUD model to optimize the target and OAR doses. The results showed that the fEUD model can effectively evaluate plans for brain, head and neck, lung, pancreas and prostate tumours. In our previous study, the fEUD model was successfully applied to evaluate the quality of the physical scheme in cervical cancer. The fEUD is computed according to the following equation:


$$\text{fEUD}=1/\left[1+k\cdot \frac{\sum_{i=1}^{n}\omega_{i}\cdot {\text{EUD}}_{\text{OAR}}^{i}}{\sum_{j=1}^{m}\omega_{j}^{\prime }\cdot {\text{EUD}}_{\text{Target }}^{j}}\right]$$

where *n* and *m* are the numbers of OARs and targets, respectively, *w*i and *wj* are the corresponding weighting factors, and *k* is the relative importance factor between the weighted sums of the EUDs for all targets and the OARs. We set *wi*, *wj* and *k* to 1 in this study. The fEUD value ranges from 0 to 1, with greater values indicating superior plan quality. Then, the EUD value is substituted into the fEUD formula to calculate the fEUD value of the prone position and supine position. Finally, we calculated fEUD_(prone-supine)_ to compare the overall quality of the two plans. A positive value of fEUD_(prone-supine)_ indicates that the prone position plan is better, and a negative value indicates that the supine position plan is better.

### Statistical analysis

Dosimetric parameters were examined by the paired t test or Wilcoxon signed-rank test. Correlations were measured using Spearman’s correlation coefficient (rho). Receiver operating characteristic (ROC) curve analyses were used to examine the predictive validity of the somatotype parameters. Linear regression models were used to explore more conveniently measurable predictors. All statistical analyses were conducted by SPSS Statistics software for Windows ver. 25.0 (IBM Corp., Armonk, NY). Differences were considered significant at *p* values < 0.05.

## Results

### Dosimetric analyses

Between June 2020 and June 2021, 160 female patients underwent whole-breast RT after BCS were randomly chosen for this study. Of these patients, 58 did not meet the inclusion criteria, and 39 did not give consent and were excluded. Finally, a total of 63 patients were enrolled (33 with left-sided and 30 right-sided breast cancer). The baseline patient characteristics are shown in **Table 1**.

**Table 1:** Patient and tumor characteristics (N=63).

Characteristic	NO. (%)	Mean	Median	Range
Age (year)		48	48	23-70
BMI (kg/m^2^)		23.85	23.15	18.73-32.45
BSA (m^2^)		1.69	1.73	1.49-2.05
Bust size (cm)		91.33	90.50	73.50-120.00
Chest size (cm)		82.84	79.75	68.00-98.50
CTV (cm^3^)				
Supine position		549.24	553.38	129.49-1916.30
Prone position		595.67	661.10	130.04-1823.37
Side				
Left	33(52.38%)			
Right	30(47.62%)			
Quartant				
medial-upper	14(22.22%)			
medial-lower	2(3.17%)			
lateral-upper	37(58.73%)			
lateral-lower	10(15.87%)			
Breast shape				
Disc-type	31(49.21%)			
Cone-type	6(9.52%)			
Drop-type	25(39.68%)			
Hemisphere-type	1(1.58%)			
Cup size				
AA	5(7.94%)			
A	7(11.11%)			
B	29(46.03%)			
C	19(30.16%)			
D	2(3.17%)			
E	0			
F	0			
G	1(1.58%)			

BMI, body mass index; BSA, body surface area. Bust size is measured as the circumference around the chest at the plane of the nipple. Chest size is measured as the circumference around the chest under the fold of the breasts .Breast volume measured by CTV (clinical target volume),in unit of cm^3^.

We performed comparisons between the prone and supine positions for the entire patient cohort, and the results are summarized in **Table 2**. For all patients, the prone position reduced the doses to lungs but increased the average volume of the breast and ipsilateral lung and the Dmean of the contralateral breast relative to the supine position (p< 0.05). For left-sided BC, compared with those of the supine position, all dose values (Dmean and V5-V40) of the heart and the Dmax and Dmean of LADCA were higher in the prone position (p ≤ 0.001). For right-sided BC, the Dmax and Dmean of the LADCA was lower in the prone position than in the supine position (p< 0.001). The Dmean of the heart was lower in the prone position, although the difference was not significant.

**Table 2:** Comparison between supine and prone positions for left-sided and right-sided groups.

Variable	Left-side			Right-side		
	Supine	Prone	P-value	Supine	Prone	P-value
Volume (cm3)						
CTV	585.44±343.52	631.93±337.47	0.009	509.42±287.42	555.78±280.68	0.014
Ipsilateral lung	1084.34±210.98	1217.28±218.37	0.000	1391.08±229.38	1501.19±248.41	0.000
Heart	540.24±95.30	543.63±103.88	0.794	593.52±93.27	585.02±93.82	0.568
Contralateral lung	1338.88±220.13	1443.73±244.77	0.001	1125.61±221.58	1251.81±218.3	0.000
Contralateral breast	593.66±366.86	661.04±335.47	0.004	470.63±349.55	588.76±334.77	0.000
Target dose						
Dmean (cGy)	5188.08±37.96	5081.25±805.53	0.004	5184.48±31.48	5200.95±39.86	0.098
CI	0.02±0.02	0.02±0.02	0.201	0.02±0.01	0.02±0.01	0.688
HI	0.12±0.04	0.26±0.12	0.000	0.10±0.03	0.10±0.02	0.393
Dose in OARs						
Ipsilateral lung						
Dmean (cGy)	1103.66±835.45	552.84±119.24	0.000	932.30±159.20	643.52±153.67	0.000
V5(%)	51.67±13.44	26.27±6.56	0.000	47.75±10.08	25.09±5.59	0.000
V10(%)	25.64±6.01	10.59±4.43	0.000	25.43±5.38	13.61±4.20	0.000
V20(%)	13.60±4.10	5.82±3.19	0.000	13.71±3.91	8.80±3.30	0.000
Heart						
Dmean (cGy)	309.46±42.67	631.57±126.56	0.000	222.05±60.46	209.34±35.7	0.428
V5(%)	9.07±2.74	26.31±10.88	0.000	3.90±6.66	4.02±3.24	0.472
V10(%)	1.24±0.50	13.10±5.62	0.000	0.28±1.26	0.73±0.61	0.000
V20(%)	0.40±0.33	4.73±2.50	0.000	0.00±0.00	0.06±0.08	0.000
V30(%)	0.15±0.16	2.25±1.69	0.000	0.00±0.00	0.01±0.02	0.018
V40(%)	0.03±0.04	0.43±0.67	0.001	0.00±0.00	0.00±0.01	0.317
LADCA						
Dmin (cGy)	262.51±67.26	298.32±100.30	0.098	182.23±143.69	137.88±19.53	0.092
Dmax (cGy)	2237.98±1303.01	3314.06±1116.54	0.001	414.54±159.77	267.4±120.76	0.000
Dmean (cGy)	656.36±434.15	1459.67±1940.99	0.000	249.72±85.10	169.06±37.23	0.000
Contralateral lung						
Dmean (cGy)	349.38±114.41	184.69±57.98	0.000	272.26±97.93	115.4±14.49	0.000
V5 (%)	21.74±13.95	4.23±6.49	0.000	13.51±11.56	0.09±0.16	0.000
V10 (%)	2.57±2.76	0.86±2.82	0.001	1.18±1.31	0.00±0.00	0.000
V20 (%)	0.05±0.14	0.30±1.66	0.306	0.00±0.01	0.00±0.00	0.157
Contralateral breast						
Dmean (cGy)	401.04±112.35	578.70±202.30	0.000	361.78±107.37	526.69±142.31	0.000
V5 (%)	20.87±13.88	50.80±15.79	0.000	19.56±14.76	28.64±10.35	0.012
Liver						
Dmean (cGy)	158.68±62.20	132.13±78.81	0.085	341.73±136.47	368.23±173.58	0.428

Values are presented as mean±standard deviation. CTV, clinical target volume; LADCA, left anterior descending coronary artery; Dmin, minimum dose; Dmax, maximum dose; Dmean, mean dose; CI, conformity index; HI, homogeneity index; OARs, organs at risk; V_X_, percentage of the volume that receives more than X Gy.

### Overall plan figure-of-merit (fEUD)

**Table 3** shows the fEUD values for the prone and supine VMAT plans. We found that 19 patients (5 with left-sided and 14 with right-sided BC) benefitted from the prone position according to this quality score. The mean, minimum, maximum volume of the CTV for these 19 patients were found to be 686.45cm^3^, 396.98cm^3^, 1512.25cm^3^, respectively.

**Table 3:** fEUD values for prone plans superior to supine plans.

NO.	Side	Prone fEUD	Supine fEUD	fEUD (prone-supine)	Supine-CTV (cm^3^)
1	right	0.112893084	0.110414396	0.002478688	415.983
2	right	0.096232005	0.094488975	0.001743031	521.241
3	right	0.120423487	0.091917231	0.028506255	1916.304
4	right	0.091174089	0.089517571	0.001656518	396.978
5	right	0.085721694	0.067475742	0.018245952	524.844
6	left	0.079840625	0.072586274	0.00725435	1512.249
7	left	0.073865475	0.057064462	0.016801013	801.381
8	left	0.105543209	0.089540692	0.016002517	498.615
9	right	0.085468039	0.081230088	0.004237951	496.971
10	right	0.129498717	0.004597929	0.124900788	516.255
11	right	0.091305729	0.001192998	0.090112731	529.179
12	right	0.083498608	0.079928729	0.003569879	623.703
13	right	0.081862464	0.03907797	0.042784494	595.074
14	right	0.089141925	0.085371361	0.003770564	429.055
15	left	0.073702277	0.069422302	0.004279975	1132.620
16	right	0.080514008	0.075874783	0.004639224	515.070
17	left	0.074863464	0.067153439	0.0077100243	578.550
18	right	0.1293878265	0.123577148	0.0058106785	518.390
19	right	0.0924156387	0.083213006	0.0092026320	520.080

19/63 cases were determined as prone-position benefited according to fEUD scores’ comparison. The higher the fEUD value, the better the overall quality of plans.Supine-CTV,clinical target volume in supine computed tomography.

### Correlation analysis

**Figure 6:**
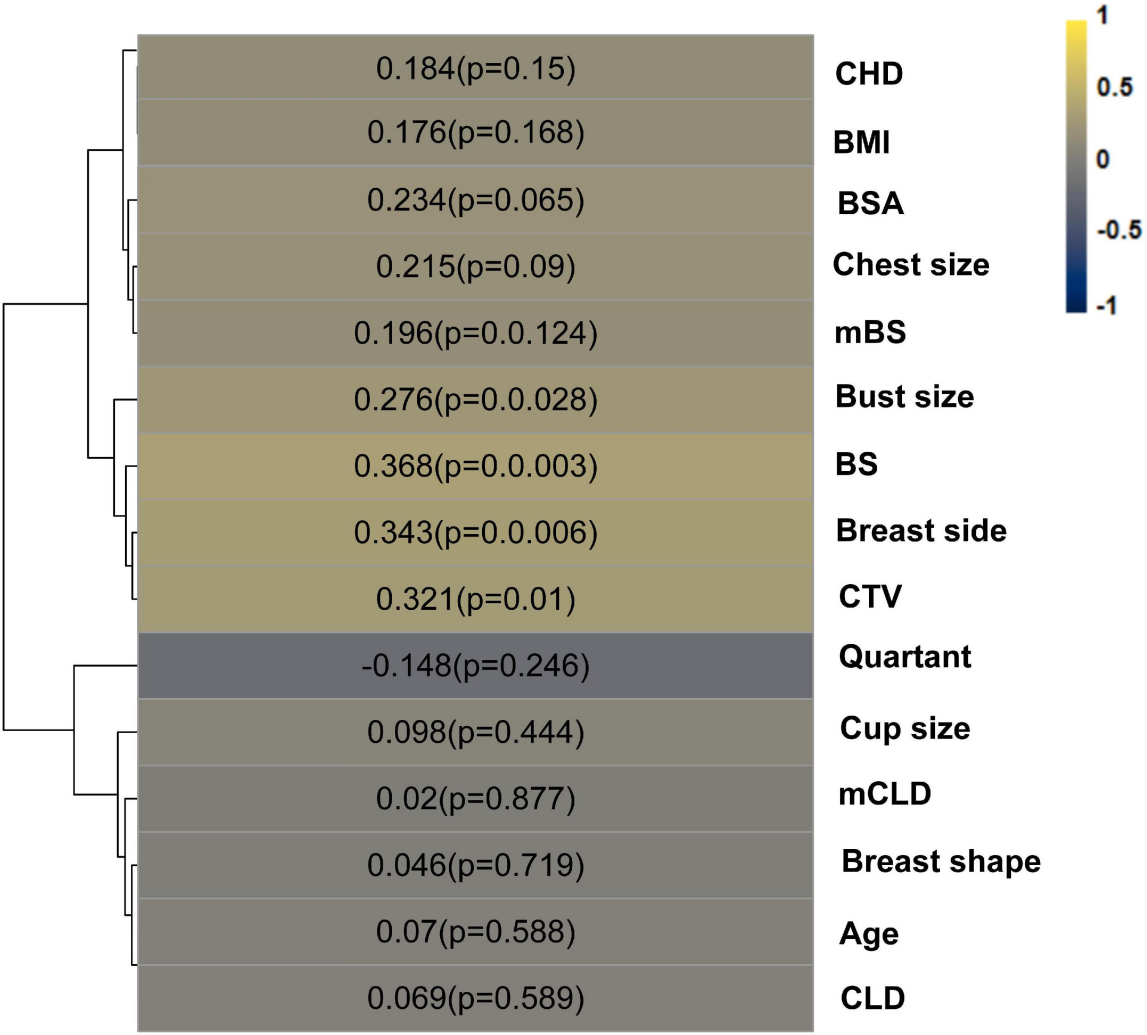
Color map of rho between “△fEUD” and somatotype parameters. “△fEUD”, whether the prone plan is better than the supine, yes=1, no=0.

**Figure 7:**
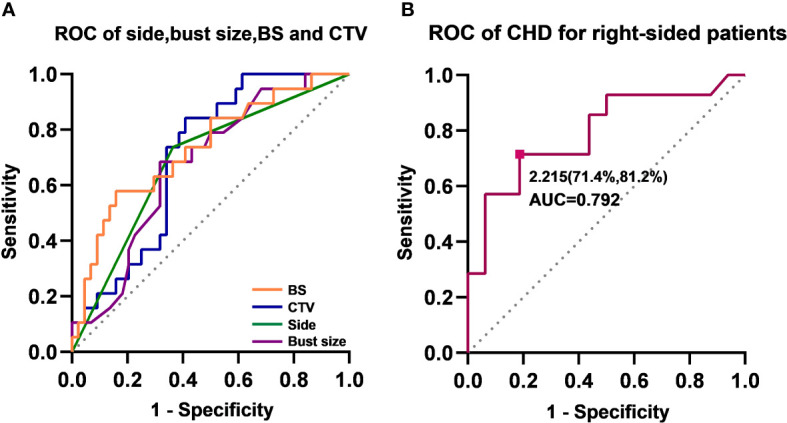
Receiver operating characteristic (ROC) curves. **(A)** ROC curves of side, bust size, BS and CTV. Area under the curve (AUC) of BS (orange), supine-CTV (blue), breast side (green) and bust size(purple) were 0.731, 0.702, 0.687, 0.673, respectively. **(B)** ROC curves of CHD for right-sided patients. The cut-off value is 2.215, with a sensitivity of 71.4% and a specificity of 81.2%.

According to the comparison between the two setups’ fEUD values, we used “△fEUD” to assess whether the prone plan was better than the supine plan; if so, the patient was given a value of 1, and otherwise. Correlations between various analysed parameters were calculated using the Pearson test or Spearman rank test, depending on the normality of the distribution. If the assumption of normality was not fulfilled, we calculated the Spearman correlation coefficients. So Spearman’s correlation analysis was conducted between the △fEUD value and the values of the different somatotype parameters (**Figure 6**). **Figure 6** shows the correlation between somatotype parameters and the △fEUD value; for example, the value in the BS grid indicates that the Spearman correlation coefficient (rho) between BS and △fEUD is 0.368, and the corresponding p value is 0.003. We found a weak, positive correlation between BS and △fEUD, and the p value indicates statistical significance. In other words, a longer BS indicates a greater likelihood that the prone position will be better than the supine position. △fEUD was weakly negatively correlated with breast side, bust size, BS and CTV (rho=0.276~0.368, p< 0.05). Subsequently, a multi-index ROC curve was drawn to evaluate the accuracy of these predictors. As shown in **Figure 7A**, the AUC values for supine CTV, BS, bust size and breast side were 0.702, 0.731, 0.673 and 0.687, respectively; this indicated that supine CTV≥495.996 cm^3^ (68.4% sensitivity, 68.2% specificity), BS≥21.735 cm (57.9% sensitivity, 84.1% specificity), bust size≥92.25 cm (84.2% sensitivity, 59.1% specificity) and breast side=right (73.7% sensitivity, 63.6% specificity) could predict a benefit from the prone position.

The above results potentially suggest that right-sided breast cancer patients with a CTV≥495.996 cm^3^, BS≥21.735 cm and bust size≥92.25 cm were very likely to benefit from prone RT. However, the CTV and BS values were not available directly from routine chest CT images. Therefore, we attempted to explore the relationship between BS and CTV and other directly measurable somatotype parameters. Positive correlations were identified between BS and breast shape (rho=0.468, p< 0.001) and between CTV and cup size (rho=0.452, p< 0.001), according to the Spearman correlation analysis. Analysis of the linear models (**Table 4**) demonstrated that BS≥21.735 cm could represent a breast shape of at least drop type. The model-dependent variable was the BS (linear variable). The independent variable was breast shape (categorical variable), including drop-type, hemisphere-type, cone-type and disc-type, as listed in **Table 1**. **Table 5** shows that CTV≥495.996 cm3 could represent a cup size of at least B. The model-dependent variable was the CTV (linear variable). The independent variable was cup size (categorical variable), including AA, B, C, and G, as listed in **Table 1**.

**Table 4:** Coefficients of Model BS.

Model BS	Unstandardized coefficients		Standardized coefficients	T	Sig.
	Beta	Std. error	Beta		
Constant	21.926	0.397	–	55.275	0.000
Hemisphere-type	-2.936	2.023	-0.164	-1.452	0.152
Cone-type	-1.901	0.902	-0.249	-2.108	0.039
Disc-type	-2.400	0.533	-0.536	-4.501	0.000

Constant: Drop-type. The dependent variable is the BS. The independent variable were breast shapes (including drop-type, hemisphere-type, cone-type and disc-type).

**Table 5:** Coefficients of Model CTV.

Model CTV	Unstandardized coefficients		Standardized coefficients	T	Sig.
	Beta	Std. error	Beta		
Constant	511.408	46.09	–	11.096	0.000
AA cup	-283.381	120.189	-0.243	-2.358	0.022
A cup	-60.683	104.523	-0.060	-0.581	0.564
C cup	134.293	71.119	0.201	1.888	0.064
G cup	1404.896	252.446	0.557	5.565	0.000

Constant: B cup. The dependent variable is the CTV. The independent variable were cup sizes (including AA, B, 291 C, and G cup).

Lower doses were delivered to the heart, LADCA and both lungs for right-sided breast cancer patients, and the fEUD model scored 14/30 right-sided breast cancer patients as the “prone beneficial group”, as previously described. Based on these data, we found that the CHD was significantly and positively associated with △fEUD among right-sided breast cancer patients (rho=0.506, p =0.004), and ROC curve analyses showed an AUC of 0.792 (**Figure 7B**). When using 2.215 cm as the cut-off value, the CHD index achieved a sensitivity of 71.4% and a specificity of 81.2% in predicting a successful response to prone RT for right-sided breast cancer patients. The CHD was originally designed as a cardiac dose predictor; Spearman’s correlation analysis showed that the CHD was negatively correlated with ΔHeart V10 (prone-supine) among right-sided BC patients (rho=-0.441, p< 0.05) but was not correlated with the heart dose values among left-sided BC patients.

## Discussion

Prone-position breast RT has previously been confirmed to be more beneficial for women with pendulous or large breasts of volumes ≥750 or 920.3 cm^3^ than the supine position (6, 8) because it elongates the treated breast away from the chest wall, which could help to prevent acute skin toxicity, especially along the inframammary fold. This study is one of few about prone breast RT that focus specifically on patients of Eastern ethnicities, such as Chinese, Korean and Japanese, who usually have a smaller breast size and body size than Western women.

Our results suggest that right-sided BC patients with a bust size≥92.25 cm, drop-type breasts and cup size≥ B are highly likely to benefit from prone positioning, while left-sided BC patients conversely are unsuitable for prone RT because of their higher heart and LADCA doses than in the supine position. According to relevant previous studies, the reasons for this phenomenon may include the following. 1) The heart could fall anteriorly towards the chest wall due to gravity in the prone position, moving it closer to the breast target volume and increasing the area that receives higher doses. 2) The average breast size was 549.24 cm^3^ (in the supine position) in this research, generally smaller than the recommended prone-beneficial breast volume of 750 cm^3^ in some studies (6). Taking the motion of the heart into account, if the breast is not sufficiently large and pendulous enough to be pulled away from the chest wall, the cardiac dose is likely to increase. 3) The RT technique used in this study is VMAT. Compared with IMRT, which was used in the majority of previous prone-position breast RT studies, the VMAT technique has been shown to improve the target dose homogeneity and conformity but inferior in terms of cardiac protection (15, 25). Our institution has been using the VMAT technique for many years for BC patients who receive RT after BCS in the supine position. With the goal of ensuring better target area coverage, there have been ongoing measures and concerted efforts to help reduce the cardiopulmonary dose as much as possible. Nevertheless, the possible benefit from prone RT for left-sided BC patients cannot be completely excluded. Our research found that the minimum CTV of left-sided BC patients in the prone-beneficial group was 498.615 cm^3^. A Korean study (11) also showed a dosimetric advantage in prone breast RT for patients with a small breast size (approximately 100 cm^3^).

When exploring the relationship between body shape and dosimetry, we chose two methods to collect somatotype parameters, i.e., anthropometric and image CT measurements. Moreover, the fEUD model, proposed by Qi et al. (24) was used to score the prone and supine plans for a quantitative assessment of overall quality. The OARs in the formula do not include the skin, spinal cord, or oesophagus, which are less irradiated within the treatment field. Correlation and ROC curve analyses showed that the possibility of a benefit from the prone position increased for a CHD≥2.215 cm for right-sided BC patients.

Several studies (20, 21, 26) have demonstrated that the maximum heart distance (MHD) is a good predictor of the mean heart dose. The MHD was measured as the maximum width of the heart in the tangent fields. Nonetheless, considering the following limitations of the MHD, we did not use it in this study. 1) The MHD needs to be recorded on beam’s eye view of the simulation CT, not on a routine physical examination CT. 2) BC can be either left or right-sided, the MHD in this study was not always a positive value but could also be 0 or negative. Therefore, it cannot be comprehensively and efficiently measured and analysed. 3) The central level of the heart is the distance to the level where the MHD is located, and there is no clear relationship between the two (27). In addition, although it has been demonstrated that other CT lines, such as BS, CLD, mBS and mCLD, are related to cardiopulmonary sparing, they do not yield an obvious prediction.

Therefore, we creatively defined the CHD, which is longer in the prone position than in the supine position because of the left-anterior motion caused by gravity. Logically, if a left-sided BC patient has a longer CHD in the supine position, it means the heart is closer to the target area, and the irradiated volume and dose to the heart will increase when changing from the supine to the prone position. In contrast, the longer the CHD is, the more cardioprotective it is for right-sided BC patients. Consistent with the above hypothesis, our results indicate that the CHD was a good predictive parameter that could be measured on routine chest CT to help select patients with right-sided BC who may benefit from prone-position radiotherapy.

The clinical application and popularization of prone breast RT are mainly restricted for the daily repeatability and stability. Some patients can not tolerate RT in the prone position, especially those with lumbar spine diseases or thoracic malformations. In studies concerning prone BC RT, multiple institutions have modified their prone setups to improve comfort and reduce errors (28). At present, there is no standardized prone-treatment board for breast RT. The prone boards from Orfit, Bionix, and especially Civco have been described in related studies (11, 29). Our prone board was provided by Klarity, and the tendency of the heart to move left anteriorly was less obvious, but the separation of the contralateral breast from the tangential field was not as notably protective as with the board from Civco. No comparison related to comfort and stability could be made.

We first raised the conception of CHD in this study to compare prone vs. supine whole breast radiotherapy for Chinese women, whose somatotype is relatively smaller than that of Western women. We sought to determine whether the smaller body figures and breast size of the Chinese population could benefit from prone radiotherapy. Additionally, we attempted to identify that anatomical characteristics could potentially indicate the benefit of normal tissue, further select the dominant treatment position without two CT simulations,which means more costs for the patients and more workload for physicians and physicists. We also used fEUD models in a innovative and prudent manner to quantitatively evaluate the overall merits of the two plans and the CHD and other geometric lines to explore their correlation with dosimetry.

However, we are aware that the relative small number of cases might increases the contingency of our analysis and some associations might be underestimated. Further studies in a wider cohort are needed to validate our existing results in a greater depth.

## Conclusions

For whole-breast irradiation after breast-conserving surgery, compared with the supine position, the prone position resulted in lower heart and ipsilateral lung doses for right-sided BC patients, while higher heart and LADCA doses were observed for patients with left BC. The prone benefit was more prominent for right-sided BC patients with drop-type breasts, greater bust and cup sizes, and, notably, longer CHD.

## Data availability statement

The raw data supporting the conclusions of this article will be made available by the authors, without undue reservation.

## Ethics statement

All patients were asked to provide their written informed consent before being registered in the study, and the present study was approved by the ethics committee of Tumor Hospital of Yunnan Province (approval number of Institutional Review Board: KYLX2022025). The patients/participants provided their written informed consent to participate in this study.

## Author contributions

Conceived and designed the analysis: YG, HB, LL, YX, LW, YH. Collected the data: LW, WL, YH. Contributed data or analysis tools: LW, LL, LC, Performed the analysis: YG, HB, XP, LC, YX, Wrote the paper: YG, WL, YH. All authors contributed to the article and approved the submitted version.

## Funding

This research was supported by the Cancer research program of National Cancer Center of China(NCC2017A32), Ten-thousand Talents Program of Yunnan Province (Yunling scholar), Yunnan Provincial Training Funds for High-level Health Technical Personnel (No.L-2018001).

## Conflict of interest

The authors declare that the research was conducted in the absence of any commercial or financial relationships that could be construed as a potential conflict of interest.

## Publisher’s note

All claims expressed in this article are solely those of the authors and do not necessarily represent those of their affiliated organizations, or those of the publisher, the editors and the reviewers. Any product that may be evaluated in this article, or claim that may be made by its manufacturer, is not guaranteed or endorsed by the publisher.
